# Esophageal duplication cyst mimicking type I achalasia in an adult female: A rare case report with diagnostic challenges

**DOI:** 10.1016/j.ijscr.2025.111954

**Published:** 2025-09-18

**Authors:** Mohammad Alaa Aldakak, Abdulkader Mehli, Nizar Alabdullah, MHD-Fadi Alshurbaji, Eias Abazid, Abdulghani Alshalabi

**Affiliations:** a Faculty of Medicine, Damascus University, Damascus, Syrian Arab Republic; b Al Assad University Hospital, Faculty of Medicine, Damascus University, Damascus, Syrian Arab Republic

**Keywords:** Esophageal duplication cyst, Pseudoachalasia, Dysphagia, Achalasia mimic, Case report

## Abstract

**Background:**

Esophageal duplication cysts (EDCs) are rare congenital anomalies typically diagnosed in infancy. Adult presentations are uncommon and may mimic other esophageal pathologies, including motility disorders such as achalasia, posing diagnostic challenges.

**Case presentation:**

We report the case of a 45-year-old Arab female with a seven-year history of progressive dysphagia, halitosis, and retrosternal burning. Radiological and manometric findings were initially suggestive of type I achalasia. However, intraoperative findings revealed an esophageal diverticulum containing purulent material, and histopathological analysis confirmed a duplication cyst lined by respiratory-type epithelium with a well-formed muscular wall. The cyst was successfully resected laparoscopically, and symptoms resolved postoperatively.

**Clinical discussion:**

Although achalasia has a well-defined manometric profile, rare structural anomalies such as duplication cysts can mimic its presentation. The absence of pathognomonic imaging findings, combined with non-specific clinical symptoms, may result in misdiagnosis. High-resolution manometry, while useful, may not distinguish between primary motility disorders and extrinsic or intramural mechanical causes. Surgical exploration remains the definitive diagnostic and therapeutic step in atypical or unresponsive cases.

**Conclusion:**

Esophageal duplication cysts should be considered in the differential diagnosis of achalasia-like presentations, especially when findings are atypical or symptoms progress despite standard evaluation. Early recognition and surgical resection are key to definitive management and symptom resolution.

## Highlights

• Esophageal duplication cysts are rare congenital anomalies that may mimic common motility disorders such as achalasia
• We report the case of a 45-year-old Arab female with progressive dysphagia, initially misdiagnosed as type I achalasia
• The lesion was ultimately identified intraoperatively as an infected esophageal duplication cyst and successfully resected via a laparoscope
• This case underscores the importance of considering structural anomalies in the differential diagnosis of achalasia-like symptoms

## 1 Introduction

Esophageal duplication cysts (EDCs) are uncommon congenital anomalies, usually diagnosed during childhood, with rare cases remaining asymptomatic until adolescence [1,2]. They arise during the first trimester of embryonic development due to defective intrauterine vacuolization of the esophageal lumen [1,2]. Their presentation varies according to cyst size and location, with possible symptoms including dysphagia, epigastric discomfort, retrosternal pain, respiratory distress, or hemoptysis. Imaging modalities such as chest radiography, esophagography, ultrasonography, computed tomography (CT), and magnetic resonance imaging (MRI) help differentiate EDCs from other benign and malignant esophageal pathologies, though preoperative diagnosis remains challenging due to non-specific findings [3]. Histologically, EDCs may be lined with alimentary squamous epithelium or tracheobronchial-type mucosa, making distinction from bronchogenic cysts difficult [2]. Untreated EDCs risk complications such as hemorrhage, infection, or malignant transformation, making surgical excision the standard treatment [1,4]. While open thoracotomy was once the preferred approach, minimally invasive thoracoscopic techniques are increasingly favored [5]. Here, we present a rare case of an esophageal duplication cyst in an adult patient, clinically and manometrically mimicking type I achalasia. The work has been reported in line with the SCARE criteria [6].

## 2 Case presentation

A 45-year-old married Arab woman, non-smoker and non-alcoholic, presented to the Emergency Department with a 7-year history of progressively worsening dysphagia to liquids and solids, accompanied by chronic halitosis and intermittent retrosternal burning. She denied odynophagia, regurgitation, vomiting, weight loss, fever, or other gastrointestinal/respiratory symptoms. Past history was otherwise unremarkable, except for two prior pilonidal sinus surgeries (latest 22 years ago) and a remote depressed skull base fracture (25 years earlier). On examination, she was well with stable vitals; cardiovascular, respiratory, abdominal, and neurological exams were normal (soft, non-tender abdomen, normal bowel sounds, no lymphadenopathy). Baseline labs (CBC, liver/renal function, electrolytes, coagulation, glucose) were within normal limits.

**Fig. 1:**
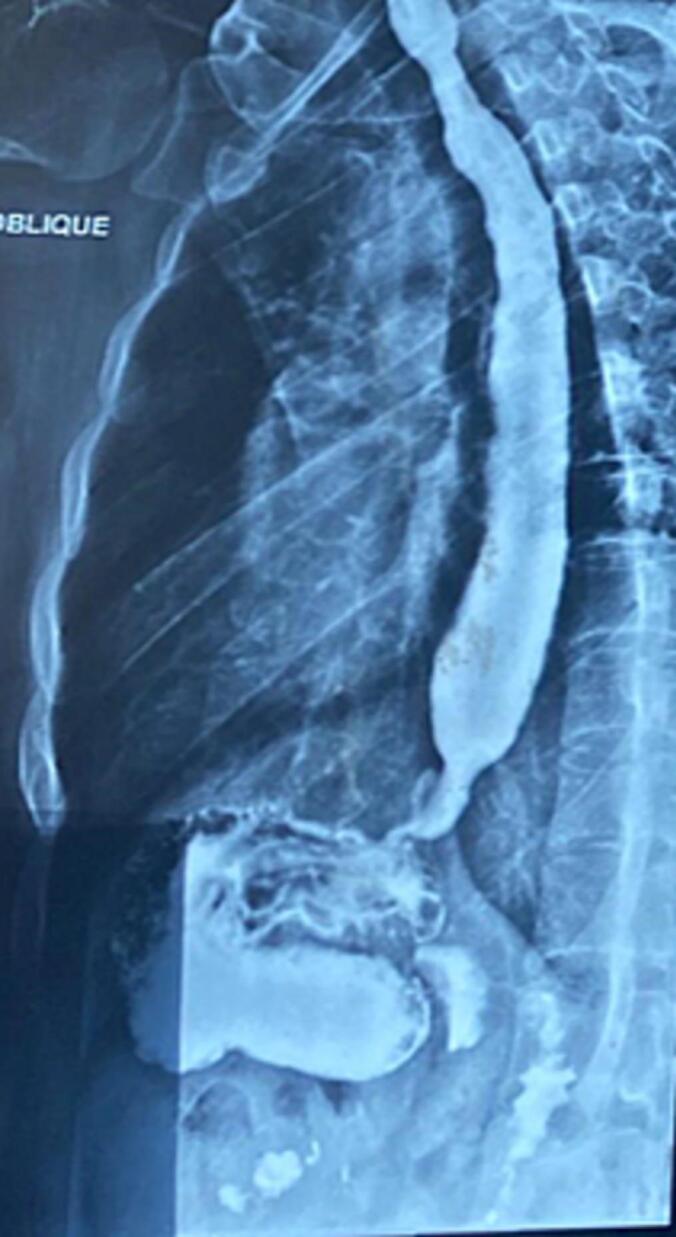
Barium swallow demonstrates a markedly dilated thoracic esophagus with multiple levels of retained contrast and a characteristic “bird-beak” tapering at the gastroesophageal junction, consistent with classic achalasia.

**Fig. 2:**
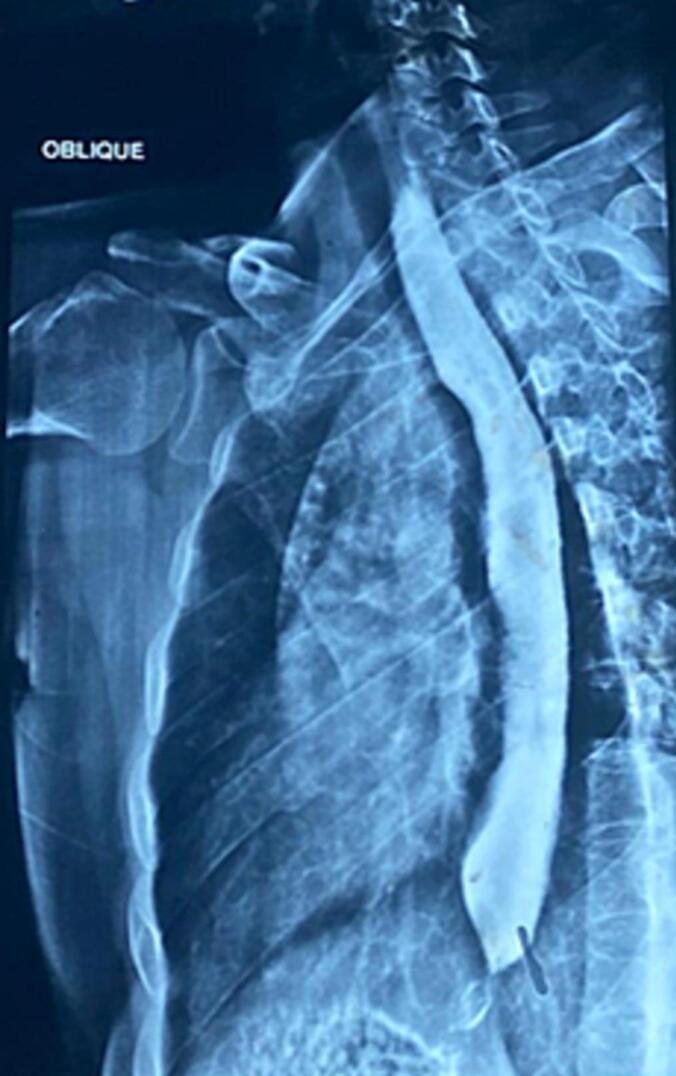
Oblique view from the barium esophagogram further illustrating the absence of esophageal peristalsis, contrast stasis, and distal tapering suggestive of impaired lower esophageal sphincter relaxation.

**Fig. 3:**
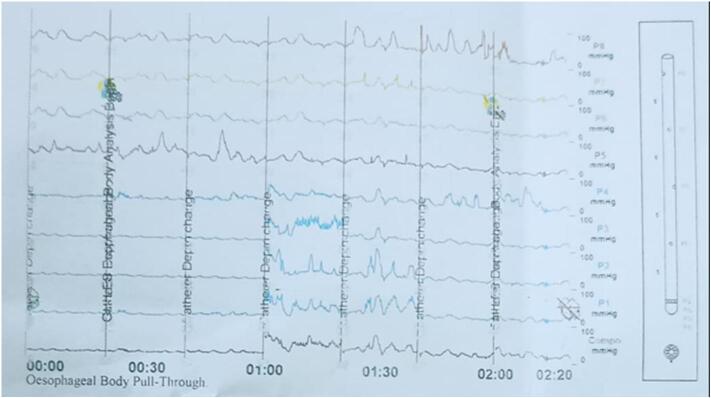
High-resolution esophageal manometry showing complete aperistalsis along the esophageal body with markedly elevated lower esophageal sphincter pressure (65 mmHg), diagnostic of type I achalasia.

A barium swallow demonstrated a dilated thoracic esophagus with multiple static contrast columns and a classic “bird-beak” narrowing at the gastroesophageal junction, along with delayed esophageal emptying and no evidence of hiatal hernia [Fig. 1, Fig. 2]. Upper gastrointestinal endoscopy showed a grossly normal esophagus with a competent cardia, patchy erythema in the gastric fundus, and a small fibrin-covered ulcer at the anterior wall of the duodenal bulb; biopsies were taken accordingly. Histopathological analysis revealed mild chronic gastritis with no dysplasia, intestinal metaplasia, or *Helicobacter pylori* infection; the esophageal mucosa was unremarkable. High-resolution esophageal manometry demonstrated complete absence of peristalsis, significantly reduced wave amplitude and propagation, and a markedly elevated lower esophageal sphincter (LES) pressure reaching 65 mmHg—findings initially suggestive of type I achalasia [Fig. 3].

**Fig. 4:**
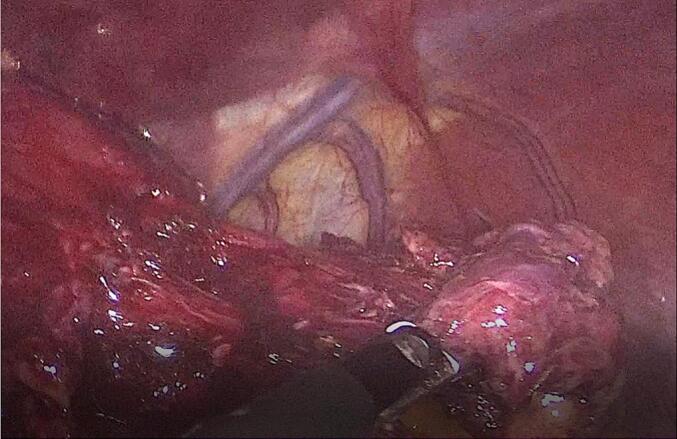
Intraoperative laparoscopic image showing the esophageal pseudo-diverticulum adjacent to inflamed tissue prior to resection.

**Fig. 5:**
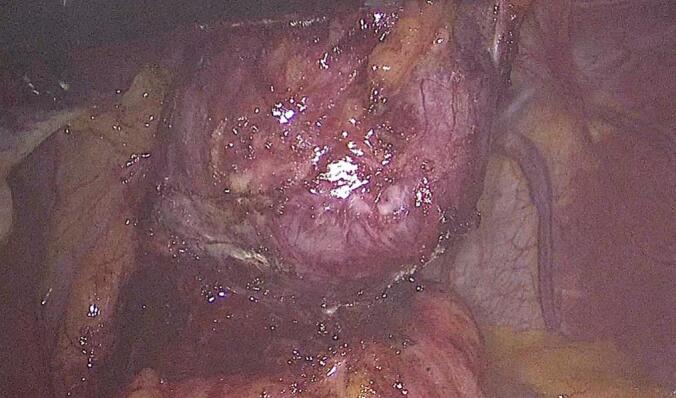
Laparoscopic view depicting the resected esophageal diverticulum containing purulent material, suggestive of superinfection within a duplication cyst.

A laparoscopic transhiatal surgical approach was undertaken, during which five trocars were inserted and an esophageal diverticulum containing purulent material was identified and resected using a 60 mm GIA stapler [Fig. 4, Fig. 5]. A leak test was negative, and a nasogastric tube was inserted. The surgery was performed by a senior consultant general surgeon with extensive experience in minimally invasive upper gastrointestinal surgery.

**Fig. 6:**
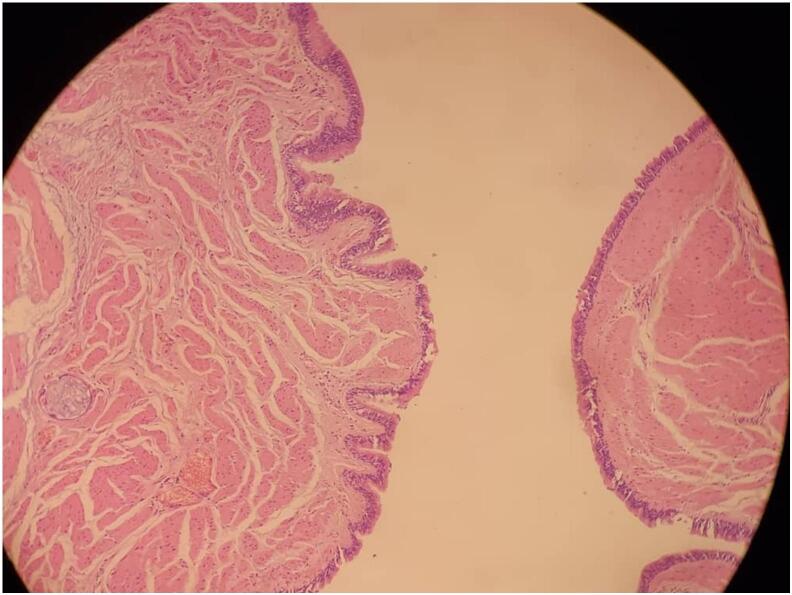
Low-power photomicrograph of the cyst wall showing a well-organized smooth muscle coat and internal lining of pseudostratified ciliated columnar epithelium (H&E stain), consistent with an esophageal duplication cyst.

**Fig. 7:**
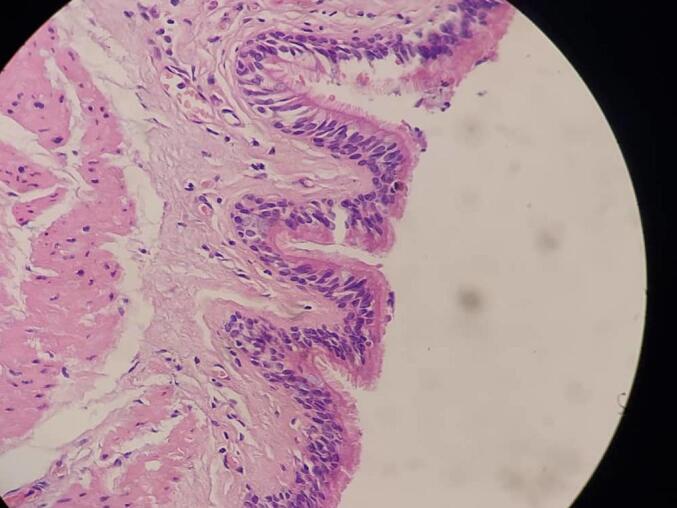
High-power histological image illustrating the respiratory-type epithelial lining of the cyst with ciliated pseudostratified columnar cells and underlying smooth muscle bundles, confirming the diagnosis of esophageal duplication cyst (H&E stain).

Histopathological examination of the resected specimen revealed a well-defined cystic wall lined by pseudostratified ciliated columnar epithelium of respiratory-type origin with an intact smooth muscle coat, consistent with an esophageal duplication cyst [Fig. 6, Fig. 7]. No evidence of dysplasia or malignancy was found.

The patient was followed up clinically for three months after surgery, with complete resolution of dysphagia and no recurrence of symptoms. Oral intake was normal, and no complications were reported.

## 3 Patient's perspective

“For many years, I struggled with difficulty swallowing and discomfort, not knowing the exact cause. I was initially told it might be a motility problem, but during surgery, the real cause was found. The recovery was smooth, and I am now free of symptoms and able to eat normally again.”

## 4 Discussion

Esophageal duplication cyst (EDC), first described by Blasius et al. in 1711, is a rare congenital anomaly with an estimated incidence of 1 in 82,000 (0.0122 %). It shows a male predominance with a reported male-to-female ratio of 2:1, accounting for approximately 10 % to 15 % of all gastrointestinal duplication cysts and 0.5 % to 2.5 % of esophageal tumors [[7], [8], [9]]. Esophageal duplication cysts are classified into three morphological types: cystic, tubular, and diverticular, with the cystic form being the most prevalent. Anatomically, approximately 60 % are located in the lower third of the esophagus, 17 % in the middle third, and 23 % in the upper third [10]. Although the exact pathogenesis remains under investigation, esophageal duplication cysts are believed to result from a failure of vacuolization of the embryonic foregut between the 4th and 8th weeks of gestation [11]. These cysts may either communicate with the esophageal lumen or remain completely isolated from it [12,13]. (EDC) may also be associated with other congenital anomalies, such as cystic bronchiectasis, intrapulmonary bronchogenic cysts, and bronchial atresia [11,14]. Histopathologically, esophageal duplication cysts are characterized by their intimate attachment to the native esophageal wall and the presence of two well-defined smooth muscle layers encasing the cyst, reflecting their embryologic origin from the alimentary tract. This muscular investment is a key distinguishing feature from bronchogenic cysts, which typically contain cartilaginous tissue or bronchial glands in their walls and lack a structured muscular coat. The epithelial lining of duplication cysts is variable and may include squamous, cuboidal, columnar, or ciliated columnar epithelium. In some cases, the presence of ectopic gastrointestinal mucosa within the cyst—particularly gastric or pancreatic tissue—can further complicate the diagnosis by mimicking other gastrointestinal anomalies, such as Meckel's diverticulum [15]. In our case, although the cyst was lined with pseudostratified ciliated columnar epithelium consistent with respiratory-type mucosa, it demonstrated a well-organized dual-layer muscular wall and was firmly adherent to the distal esophagus. No cartilaginous structures or bronchial glands were identified. These findings, along with its anatomical location and histological features, clearly supported the diagnosis of an esophageal duplication cyst rather than a bronchogenic cyst. Preoperative diagnosis typically requires multiple imaging modalities, such as computed tomography (CT) or magnetic resonance imaging (MRI) [7,16]. Nevertheless, endoscopic ultrasonography (EUS) remains the most effective diagnostic tool, as it allows clear differentiation between solid and cystic lesions and accurately identifies the esophageal wall layer involved [2,7]. Additional studies, such as barium swallow, can aid in the diagnostic process by identifying the exact location of the lesion and its spatial relationship to the esophageal hiatus [16,17]. In contrast, our patient underwent barium swallow and manometry, which suggested type I achalasia. The diagnosis of an esophageal duplication cyst was made intraoperatively and confirmed histologically. Esophageal duplication cysts are typically diagnosed during infancy, with up to 80 % of cases identified in early life, while only a small proportion of patients remain asymptomatic until adulthood [11,12]. A recent systematic review by Gonzalez-Urquijo et al., analyzing data from 97 adult patients with esophageal duplication cysts, reported that the majority (81 %) were symptomatic [16,17]. As the cyst enlarges, clinical symptoms typically develop based on its interaction with and compression of adjacent anatomical structures [13,14]. Achalasia is a rare primary esophageal motility disorder characterized by the failure of the lower esophageal sphincter (LES) to relax adequately during swallowing, accompanied by the absence of peristalsis in the esophageal body. Patients commonly present with progressive dysphagia to both solids and liquids, regurgitation of undigested food, chest pain, and significant weight loss due to impaired bolus transit and chronic esophageal stasis [18]. In our case, the patient exhibited a similar clinical presentation and even met the manometric criteria suggestive of type I achalasia. However, these findings were ultimately attributed to the compressive and obstructive effect of the enlarging duplication cyst, which functionally mimicked primary achalasia. In adults, the most common presenting symptoms of (EDC) include pain, dysphagia, and gastroesophageal reflux, whereas in children, airway obstruction and recurrent pulmonary infections are more frequently observed [7,12]. Adults may also experience weight loss, often secondary to food avoidance stemming from dysphagia-related anxiety [14,16,19]. If not diagnosed and managed promptly, esophageal duplication cysts can lead to serious complications such as perforation, intramural hematoma, infection, or even malignant transformation [13]. The only definitive treatment for esophageal duplication cysts is complete surgical excision. In the absence of surgical or anesthetic contraindications, the laparoscopic approach is generally preferred, as it is associated with fewer intraperitoneal adhesions and a shorter hospital stay compared to the conventional open technique [20]. Nonetheless, the standard and most effective method remains conventional posterolateral thoracotomy with en bloc resection of the cyst [21,22]. Minimally invasive techniques such as video-assisted thoracoscopic surgery (VATS) and robotic-assisted thoracoscopic surgery (RATS) have also been described. These approaches offer several advantages, including reduced postoperative pain, faster recovery, and improved cosmetic outcomes; however, their use is typically limited to pediatric patients and uncomplicated cases [14,23]. In cases involving large or complicated EDCs, open surgical resection remains the preferred strategy; however, in our patient, a laparoscopic approach was successfully employed [16]. Although conservative non-operative management has been described in select asymptomatic cases, complete excision remains definitive, and minimally invasive surgery can be considered in appropriately selected patients [24].

## 5 Conclusion

Esophageal duplication cysts, though rare in adults, can present with clinical and manometric features that closely mimic primary achalasia, leading to potential misdiagnosis and delayed treatment. This case highlights the importance of maintaining a high index of suspicion for structural lesions in patients with atypical or progressive dysphagia. Definitive diagnosis often requires intraoperative assessment, and complete surgical excision remains the treatment of choice. Early identification and management are crucial to prevent complications and achieve favorable outcomes.

## Author contribution

Mohammad Alaa Aldakak: Conceptualization, data collection, literature review, case analysis, and manuscript writing.

Abdulkader Mehli: Clinical data interpretation, literature review, and drafting of the discussion section.

Nizar Alabdullah: Assistance in manuscript editing, histopathology interpretation, and figure preparation.

MHD-Fadi Alshurbaji: Radiological analysis, image selection, and contributions to the introduction and discussion.

Eias Abazid: Surgical details documentation, intraoperative image acquisition, and critical revision of the manuscript.

Abdulghani Alshalabi: Review of references, formatting, final proofreading, and overall supervision.

All authors have read and approved the final manuscript

## Patient's consent

Written informed consent was obtained from the patient for publication and any accompanying images. A copy of the written consent is available for review by the Editor-in-Chief of this journal on request.

## Ethical approval

Institutional Review Board (IRB) approval is not required for de-identified single case reports or case histories, in accordance with institutional policies.

## Guarantor

The First Author: Mohammad Alaa Aldakak

Declaration of Generative AI and AI-assisted technologies in the writing process

Artificial intelligence tools (ChatGPT, OpenAI) were used to assist in language editing and improving the clarity of the manuscript. All AI-generated content was reviewed and verified by the authors to ensure accuracy and integrity, and no AI tools were used to generate or fabricate data.

## Funding

This research did not receive any specific grant from funding agencies in the public, commercial, or not-for-profit sectors.

## Declaration of competing interest

The authors declared no potential conflicts of interest concerning the research, authorship, and/or publication of this article.
